# Long intestinal tube splinting prevents postoperative adhesive small-bowel obstruction in sclerosing encapsulating peritonitis

**DOI:** 10.1186/1471-230X-14-180

**Published:** 2014-11-25

**Authors:** Min Li, Weiming Zhu, Yousheng Li, Jun Jiang, Jieshou Li, Ning Li

**Affiliations:** Department of General Surgery, Jinling Hospital, Medicine School of Nanjing University, NO 305 East Zhongshan Road, Nanjing, Jiangsu Province China

**Keywords:** Splinting, Long intestinal tube, Recurrence, Adhesive small-bowel obstruction, Sclerosing encapsulating peritonitis

## Abstract

**Background:**

Sclerosing encapsulating peritonitis (SEP) is a rare cause of small-bowel obstruction. The optimal treatment for this condition remains controversial.

**Methods:**

In this study, we performed a retrospective analysis of the data of 44 patients who underwent surgery for SEP between December 2001 and 2008 at our hospital. The long-term follow-up data of the patients were assessed for the recurrence of adhesive small-bowel obstruction (ASBO), and patient survival was assessed to evaluate the efficiency of tube splinting in the prevention of postoperative ASBO.

**Results:**

Of the 44 patients who underwent surgery for SEP, 33 underwent simple enterolysis along with tube splinting, while the remaining underwent only simple enterolysis. The median follow-up period was 79.4 ± 24.8 months (range: 8–123 months). The rate of complications was 9.1% and 6.1% in the simple enterolysis group and tube-splinting group, respectively (P = 0.73). The recurrence rate of ASBO was lower in the tube-splinting group (6.7%) than in the simple enterolysis group (40%) (P = 0.02).

**Conclusion:**

Our findings indicate that tube splinting may be more useful than simple enterolysis alone in preventing the recurrence of ASBO in patients with SEP.

## Background

Sclerosing encapsulating peritonitis (SEP), a rare cause of intestinal obstruction, is characterized by the formation of a fibrotic membrane that either partially or completely encases the small bowel [1]. The etiology and pathogenesis of SEP are yet to be elucidated. The salient clinical features of SEP are recurrent episodes of acute or subacute small-bowel obstruction (SBO); weight loss; nausea; anorexia; and, occasionally, a palpable abdominal mass [2]. SEP is difficult to diagnose and is often detected during surgery for the correction of SBO. The incidence of postoperative adhesive SBO (ASBO) has been reported to be 14.3–29% [1, 3].

SEP is a rare disease and most studies on this condition are based on a small number of patients and short follow-up periods [4–7]. The optimal treatment strategy for SEP remains controversial. Although there is a general consensus on the need for surgery for the treatment of the SEP, various surgical approaches are employed, including subtotal excision of the membrane, enterolysis, small-bowel resection, and exploratory laparotomy, followed by postoperative medical treatment [3, 8, 9].

Long intestinal tube splinting, which was first described by White [10] and popularized later by Baker [11], has been proven to be efficient in mitigating the disease recurrence rate in patients with ASBO [12, 13]. However, no study has thus far examined the efficiency of this procedure in patients with ASBO due to SEP. This study is aimed at determining whether tube splinting in patients with SEP is more effective than simple enterolysis alone in preventing the recurrence of ASBO over the long term.

We analyzed the data of 44 patients with SEP who were followed up for a mean of 79.4 months. To our knowledge, this study has the largest sample size and longest follow-up duration recorded thus far among investigations on patients with SEP.

## Methods

### Study protocol

The study was designed as a retrospective investigation of the long-term follow-up data of all patients who had undergone surgery for the correction of SBO due to SEP at the Department of General Surgery, Jinling Hospital, Medicine School of Nanjing University, China, between December 2001 and 2008. The study protocol was approved by the Ethics Committee at Jinling Hospital and was in compliance with the Chinese national legal regulations.

### Patients

The diagnosis of SEP was confirmed on the basis of intraoperative findings, as described previously [1]. The patients were classified into two groups depending on whether they underwent simple enterolysis alone (simple enterolysis group; n = 11) or simple enterolysis along with long intestinal tube splinting (tube splinting group; n = 33). The diagnosis of postoperative ASBO, as per the medical records, was defined by hospital admission with abdominal pain, distention, vomiting, and obstipation (failure to pass gas and feces) along with radiological evidence of intestinal obstruction. The demographic characteristics and medical records of the 44 patients were carefully reviewed for the assessment of postoperative morbidity and hospital mortality. All patients were followed up by telephonic interviews to evaluate the recurrence rate of ASBO. The final follow-up interviews were held on December 30, 2012. Survival was calculated as the duration between the date of the operation for ASBO to the date of recurrence, subsequent abdominal operation, death, or the end of the study.

### Statistical analysis

The demographic and baseline characteristics of the patients were presented in terms of mean ± standard deviation. The recurrence rate of ASBO was determined by survival analysis methods. The cumulative survival rates were calculated by the Kaplan–Meier method, and the intergroup differences were assessed using the log-rank test. Relative risks were expressed as hazard ratios (HRs) with a 95% confidence interval (CI). The complication rates in the two groups were compared by the chi-square test. Statistical analysis was performed using SPSS 18.0 for Windows (SPSS Inc., Chicago). A P value of <0.05 was considered statistically significant.

## Results

Between December 2001 and 2008, 44 patients (men: 36; women: 8; median age: 44 ± 11.5 years; range: 19–70 years) underwent surgical correction of ASBO due to SEP. Thirty of these patients had a history of previous surgery, including nine who had undergone exploratory laparotomy for SEP. None of the patients had received peritoneal dialysis.

### Intraoperative findings

**Figure 1:**
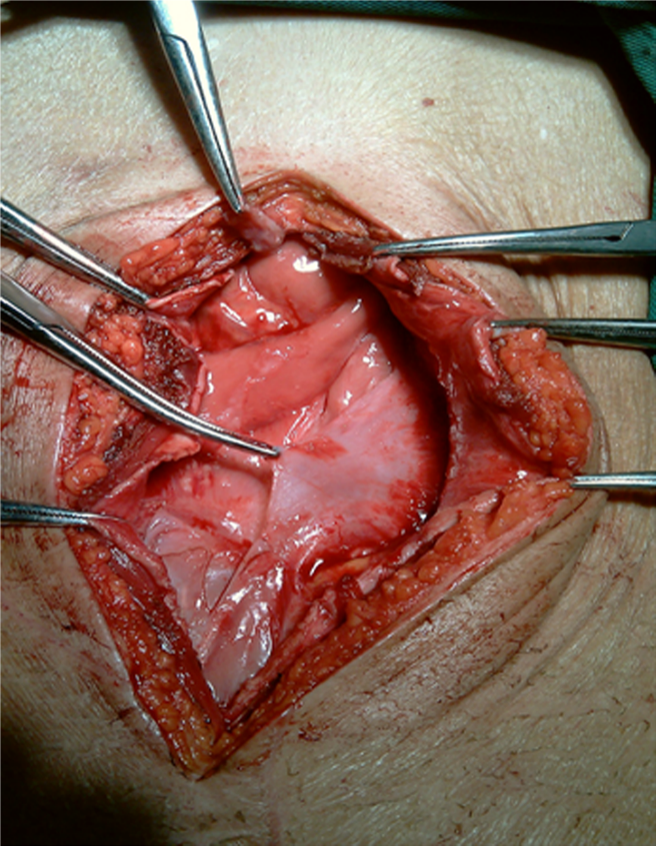
**Intraoperative photograph of a patient with SEP (membrane encapsulates the entire intestine).**

On laparotomy, 31 of the 44 patients were found to have membrane encapsulation extending throughout the entire intestine (Figure 1), while 13 had encapsulation of only small parts of the intestine. All patients had extensive adhesions between different bowel segments and between the membrane and intestine, with 12 patients exhibiting multiple matted inter-bowel adhesions. On laparotomy, all patients received total resection of membrane and lysis of adhesions. Six patients underwent inadvertent enterectomy. In all the patients, histologic examination of the membrane showed proliferation of fibrocytes and collagen fiber with nonspecific inflammatory reaction.

### Long intestinal tube splinting

**Figure 2:**
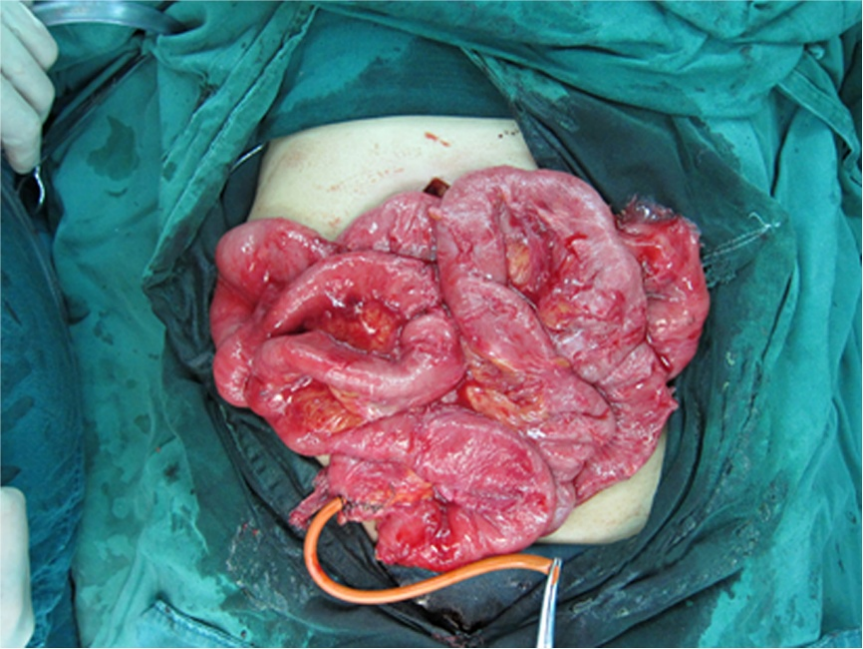
**Long intestinal tube introduced through the base of the appendix after appendicectomy.**

The decision of whether tube splinting should be performed for the patients was made on the basis of the attending surgeon’s opinion of whether tube splinting was effective in preventing the recurrence of ASBO. Therefore, some patients (n = 33) underwent tube splinting, while others did not. Two, three, or four 16-F tubes of length 150 cm were used as necessary and sutured together using 2/0 silk. Small side holes were cut at 15-cm intervals along the tubes. Appendicectomy was performed, and the tube was introduced into the base of the appendix (n = 26) or the stump of the terminal ileal anastomosis (n = 1) and then advanced upwards into the duodenojejunal flexure (Figure 2). In the remaining 6 patients who had previously undergone appendectomy, the tubes were introduced via cecostomy. The tubes were left in situ for a median duration of 11.5 ± 2.4 days, after which the tubes were removed.

### Mortality and morbidity

No case of postoperative death was recorded in this cohort. Three patients developed early postoperative inflammatory intestinal obstruction, which was resolved by expectant treatment. The overall rate of complications was 6.8% (Table 1), and the rates in the simple enterolysis group (9.1%) and the tube splinting group (6.1%) did not show any significant difference (P = 0.73).

**Table 1:** **Postoperative complication and ASBO in two groups**

	n	Complication (%)	Follow-up (n)	ASBO (n) (%)
Enterolysis group	11	1(9.1%)	10	4(1 re-operation) (40%)
Tube splinting group	33	2(6.1%)	30	2(1 re-operation) (6.7%)
Total	44	3(6.8%)	40	6(2 re-operation) (15%)

ASBO: adhesive small bowel obstruction.

### Follow up and postoperative ASBO

Four patients (1 in the simple enterolysis group and 3 in tube splinting group) were lost to follow up. The overall mean follow-up duration for the remaining 40 patients was 79.4 ± 24.8 months (range: 8 to 123 months), while that in the simple enterolysis group and tube splinting group was 65.5 ± 32.8 months (range: 8–104 months) and 84 ± 20.2 months (range: 50–123 months), respectively.

**Figure 3:**
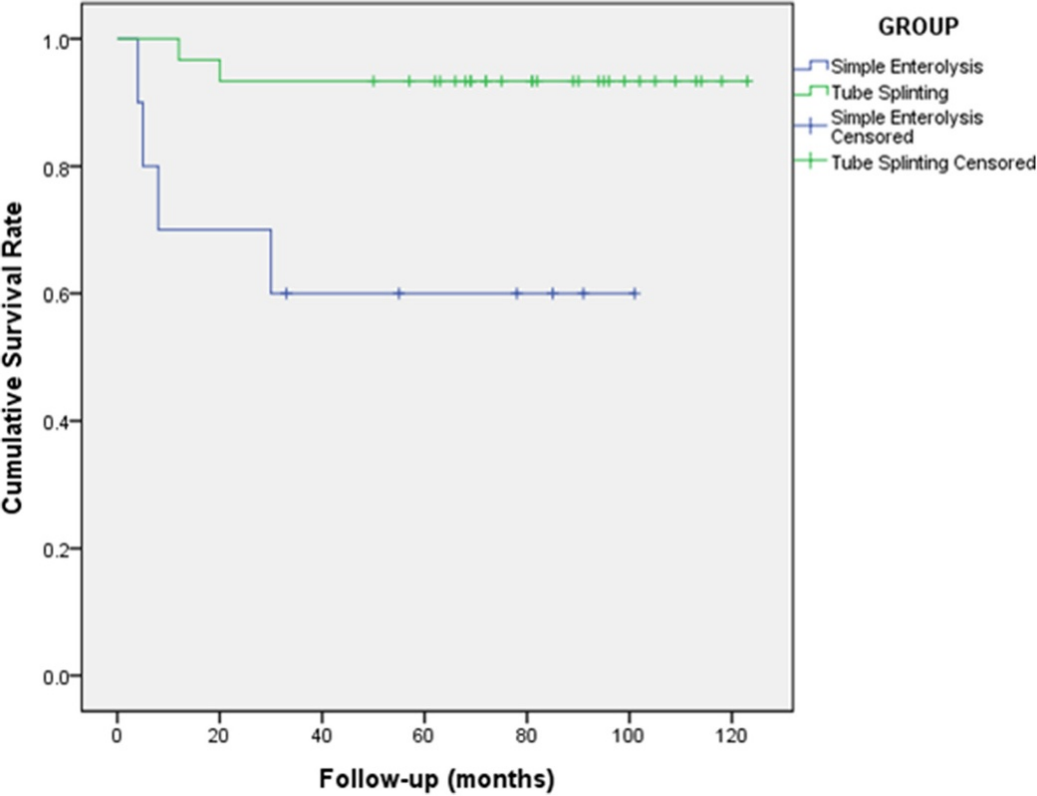
**Cumulative survival rates after surgery in patients who did or did not undergo tube splinting (Kaplan–Meier analysis).**

Six patients (15%) had 17 episodes of postoperative ASBO: 15 were managed conservatively and 2, surgically (Table 1). One patient required 1 hospital admission for postoperative ASBO, 1 required 2 admissions, 3 required 3, and 1 required 5. The cumulative incidence of ASBO was 10% at 1 year, 12.5% at 2 years, and 15% at 3 years and at the end of study. The number of postoperative ASBO episodes was greater in the simple enterolysis group (4/10; 40%) than in the tube splinting group (2/30; 6.7%) [P = 0.02; HR = 0.134 (0.024–0.773)] (Figure 3).

## Discussion

SEP is a rare clinical condition of unknown etiology. It is believed to be caused by recurrent low-grade or subclinical peritonitis, which does not manifest with any significant abdominal signs and eventually leads to sclerosis and membrane formation with subsequent formation of a cocoon [14]. Since the condition is not associated with any specific set of symptoms, most cases are diagnosed incidentally during laparotomy.

Due to the lack of long-term studies on a large number of patients with SEP, the optimal treatment for this condition remains debatable. Most of the pertinent studies published thus far have been conducted on a small number of patients (1–9 patients) and over a short follow-up period (10–25 months) [1, 5–8, 15, 16]. Celicout et al. [3] reported a case series of 32 patients who underwent four types of procedures, namely, membrane resection (n = 5), enterolysis with partial excision of the membrane (n = 12), intestinal resection (n = 7), and only exploratory laparotomy (n = 8). The recurrence rate of ASBO in their study was 29% over a mean follow-up period of 49.5 months. The current study investigated 44 patients who underwent surgery for ASBO due to SEP. Since all the enrolled patients had extensive adhesions between bowel segments and between the membrane and intestine, total resection of the membrane and adhesiolysis was performed in all the patients. In addition, 33 patients received long intestinal tube splinting. At the end of a mean follow-up period of 79.4 months, the recurrence rates of ASBO in the study population and in the tube splinting group were 15% and 6.7%, respectively. Compared to previous studies on the recurrence rates of ASBO due to SEP managed with simple enterolysis [1, 3, 5–8, 15, 16], ours indicated a lower rate in a larger cohort of patients managed with tube splinting, over a longer follow-up period.

The histological characteristics of the membranous tissue are proliferation of fibroconnective tissue and non-specific chronic inflammatory reaction [15]. This suggests that patients with SEP are prone to develop fibrin deposition. This, in turn, implies that postoperative adhesions in SEP patients would be inevitable since excessive deposition of fibrin leads to the formation of adhesions [17]. The strategy of long intestinal tube splinting is based on acknowledging the possibility of the formation of adhesions and fixing the bowel so as to ensure a favorable lie and thereby prevent the recurrence of ASBO. In patients having ASBO with multiple matted adhesions, long intestinal tube splinting has been shown to decrease the recurrence rate of ASBO [18]. Our study clearly showed that the recurrence rate of ASBO in the tube splinting group was lower than that in the simple adhesiolysis group (6.7% vs. 40%, P = 0.02).

Mortality due to ASBO has been reported to be 0–71% [3, 16, 19, 20]. There were no deaths in our case series. The postoperative complications recorded in the previous studies were enterocutaneous fistula and early postoperative intestinal obstruction, at a rate of 8.7–66.7% [3, 7, 16]. These rates are greater than those in our study, wherein the overall complication rate was 6.8%, without any significant intergroup differences (P = 0.73). We believe that correct preoperative diagnosis and careful separation of the adhesions are important to decrease the rate of complications. When the diagnosis is established before the surgery, the surgeon can be well prepared for the operation and take special precaution to avoid intestinal injury when the abdominal cavity is incised. Computed tomography is the modality of choice to confirm the diagnosis, and the characteristic findings in all cases are dilation of the small bowel in the middle of the abdomen and encasement of the bowels by a thick membrane sac [21].

The limitations of the study are that it is retrospective in nature and that it was conducted at a single center. These drawbacks can be overcome by further investigations on a wider population base.

## Conclusion

In conclusion, this was the largest study with the longest follow-up duration published thus far on patients with ASBO due to SEP. This retrospective study revealed that tube splinting may be more useful than simple enterolysis alone in preventing the recurrence of ASBO in patients with SEP. The rate of postoperative complications and recurrence of ASBO in this study were the lower than those reported thus far.

## References

[1] Foo KT, Ng KC, Rauff A, Foong WC, Sinniah R (1978). Unusual small intestinal obstruction in adolescent girls: the abdominal cocoon. Br J Surg.

[2] Kawaguchi Y, Kawanishi H, Mujais S, Topley N, Oreopoulos DG (2000). Encapsulating peritoneal sclerosis: definition, etiology, diagnosis, and treatment. International Society for Peritoneal Dialysis Ad Hoc Committee on Ultrafiltration Management in Peritoneal Dialysis. Perit Dial Int.

[3] Celicout B, Levard H, Hay J, Msika S, Fingerhut A, Pelissier E (1998). Sclerosing encapsulating peritonitis: early and late results of surgical management in 32 cases. French Associations for Surgical Research. Dig Surg.

[4] Serafimidis C, Katsarolis I, Vernadakis S, Rallis G, Giannopoulos G, Legakis N, Peros G (2006). Idiopathic sclerosing encapsulating peritonitis (or abdominal cocoon). BMC Surg.

[5] Mohanty D, Jain BK, Agrawal J, Gupta A, Agrawal V (2009). Abdominal Cocoon: Clinical Presentation, Diagnosis, and Management. J Gastrointest Surg.

[6] Narayanan R, Bhargava BN, Kabra SG, Sangal BC (1989). Idiopathic sclerosing encapsulating peritonitis. Lancet.

[7] Yang W, Ding J, Jin X, Wu H, Kuang J, Tao Z, Chu PG, Yen Y, Qiu W (2009). The Plication and Splinting Procedure for Idiopathic Sclerosing Encapsulating Peritonitis. J Invest Surg.

[8] Samarasam I, Mathew G, Sitaram V, Perakath B, Rao A, Nair A (2005). The abdominal cocoon and an effective technique of surgical management. Trop Gastroenterol.

[9] Liu HY, Wang YS, Yang WG, Yin SL, Pei H, Sun TW, Wang L (2009). Diagnosis and surgical management of abdominal cocoon: results from 12 cases. Acta Gastroenterol Belg.

[10] White RR (1956). Prevention of recurrent small bowel obstruction due to adhesions. Ann Surg.

[11] Baker JW (1959). A long jejunostomy tube decompressing intestinal obstruction. Surg Gynecol Obstet.

[12] Rodriguez-Ruesga R, Meagher AP, Wolff BG (1995). Twelve-year experience with the long intestinal tube. World J Surg.

[13] Sprouse LR, Arnold CI, Thow GB, Burns RP (2001). Twelve-year experience with the thow long intestinal tube: a means of preventing postoperative bowel obstruction. Am Surg.

[14] Maguire D, Srinivasan P, O’Grady J, Rela M, Heaton ND (2001). Sclerosing encapsulating peritonitis after orthotopic liver transplantation. Am J Surg.

[15] Xu P, Chen LH, Li YM (2007). Idiopathic sclerosing encapsulating peritonitis (or abdominal cocoon): A report of 5 cases. World J Gastroenterol.

[16] Eltringham WK, Espiner HJ, Windsor CW (1977). Sclerosing peritonitis due to practolol: A report of 9 cases and their surgical management. Br J Surg.

[17] Attard JA, MacLean AR (2007). Adhesive small bowel obstruction: epidemiology, biology and prevention. Can J Surg.

[18] Fazel MZ, Jamieson RW, Watson CJ (2009). Long-term follow-up of the use of the Jones’ intestinal tube in adhesive small bowel obstruction. Ann R Coll Surg Engl.

[19] Hollman AS, McMillan MA, Briggs JD, Junor BJ, Morley P (1991). Ultrasound changes in sclerosing peritonitis following continuous ambulatory peritoneal dialysis. Clin Radiol.

[20] Slingenmeyer A (1987). Preliminary report on a cooperative international study on sclerosing encapsulating peritonitis. Contrib Nephrol.

[21] George C, Al-Zwae K, Nair S, Cast JE (2007). Computed tomography appearances of sclerosing encapsulating peritonitis. Clin Radiol.

[CR22] The pre-publication history for this paper can be accessed here: http://www.biomedcentral.com/1471-230X/14/180/prepub

